# Microbiome characterization of a pre-Hispanic man from Zimapán, Mexico: Insights into ancient gut microbial communities

**DOI:** 10.1371/journal.pone.0331137

**Published:** 2025-10-08

**Authors:** Santiago Rosas-Plaza, Luisa Mainou, Gabriela Delgado, Rosario Morales, Ariana Aguilar-Romero, Ana E. Escalante, Rene Cerritos

**Affiliations:** 1 Posgrado en Ciencias Biológicas, Facultad de Medicina, Universidad Nacional Autónoma de México, Mexico City, Mexico; 2 Coordinación Nacional de Conservación del Patrimonio Cultural, Instituto Nacional de Antropología e Historia, Mexico City, Mexico; 3 Departamento de Microbiología y Parasitología, Facultad de Medicina, Universidad Nacional Autónoma de México, Mexico City, Mexico; 4 Instituto de Ecología, Universidad Nacional Autónoma de México, Mexico City, Mexico; 5 SECIHTI, CIBIOGEM (Comisión Intersecretarial de Bioseguridad y Organismos Genéticamente Modificados), Mexico City, Mexico; Vilnius University: Vilniaus Universitetas, LITHUANIA

## Abstract

The research of microbiome derived from mummified human remains, coprolites and paleofeces has gained significant interest over several decades, aiming to elucidate the evolution of microbial interactions and shed light on the lifestyles of past populations. In this study, we analyzed the gut microbiome of a pre-Hispanic male individual referred to as the Zimapán man, dated to 936 BP, discovered on the border between Mesoamerica and Aridoamerica. Employing high-throughput 16S rRNA gene sequencing on both the paleofeces and mummified intestinal tissue, we conducted a comprehensive characterization of the Zimapán man’s gut microbiome. The bacterial community was described, and a weighted UniFrac-based principal coordinates analysis (PCoA) was performed. The multivariate analysis incorporated microbiome samples from diverse environments, such as soils, compost, and both contemporary and ancient human gut microbiota. The analysis revealed bacterial groups associated with the human microbiome, including families such as *Peptostreptococcaceae*, *Clostridiaceae*, *Enterobacteriaceae*, and *Enterococcaceae*. Notably, a high abundance of the *Clostridiaceae* group was found, similar to those discovered in the intestinal tissue of mummies from other geographic regions. Additionally, unique groups such as *Romboutsia hominis*, exclusively isolated from human intestines and not previously reported in ancient human microbiomes, were identified. Furthermore, our analysis demonstrated that the bacterial composition did not resemble soil and compost environments. This initial characterization successfully achieved the goal of identifying bacterial groups of the gut microbiome in the Zimapán samples. Consequently, this study contributes to the ongoing expansion of knowledge regarding ancient microbiomes across diverse temporal, historical, geographical, and environmental contexts.

## Introduction

In recent years, there has been significant progress in the study of ancient samples through the analysis of DNA and other molecules, with the aim of exploring the evolutionary history of living organisms [1]. This approach encompasses the examination of remains from extinct organisms and ancient human populations, such as bones, hair, or mummified tissue, as well as archaeological artifacts, including various ancient tools. These materials can provide invaluable insights into understanding the history of our ancestors [2–3]. The integration of information from archaeological, anthropological, and molecular studies can significantly contribute to our understanding of human evolution and shed light on the context in which ancient populations lived, including the lifestyles of individuals [4,5]. This multidisciplinary approach enables a more comprehensive and detailed reconstruction of the evolutionary history of human populations, thereby contributing to a holistic understanding of our species.

Among the different types of ancient samples, coprolites, paleofeces, and in some very rare cases, mummified tissue, are considered significant sources of historical information [6]. Through these types of samples, valuable insights into the diet, diseases, and potential communities of commensal microorganisms present in the intestines of ancient populations (ancient human microbiome) can be obtained [6]. This is particularly important as it allows for a deeper understanding of the interaction between humans and the microbiome in natural environments that have not undergone significant modifications, unlike the substantial changes experienced in the globalized era [7]. While the studies of modern human microbiome have been conducted on populations maintaining lifestyles such as hunting and gathering, it is undeniable that they have been directly or indirectly affected by globalization and industrialization processes [7].

In particular, the study of the human microbiome has become a focal point of interest in the past decade, recognizing its correlation with the health status of human populations [8]. Research has demonstrated that the microbiome plays a crucial role in various vital functions, including the absorption of vitamins and protection against pathogens, among others [9]. Additionally, large-scale studies have shown that processes of urbanization have led to changes in the human gut microbiome [10]. In this context, analyzing changes across different scales of time and space becomes a key element in understanding alterations in the microbiome throughout the history of human civilization. Thus, efforts have been undertaken to explore the composition and potential functions of the ancient human microbiome from various populations living in different eras and civilizations, thereby providing a broader perspective on the human microbiome throughout history [11–16].

Through studies on the ancient human microbiome, it has been discovered that changes in the modern diet, exposure to toxic contaminants, and the continuous use of antibiotics have likely resulted in positive selection for bacterial taxa involved in specific metabolic activities within the human microbiome [17]. Similarly, a decrease in bacterial groups in contemporary human populations, such as *Treponema*, has been detected. *Treponema* is mainly reported in populations with traditional contemporary lifestyles (hunter-gatherers and agriculturalists) and in the ancient human intestinal microbiome [14,16]. On the other hand, the loss of functional genes in the contemporary human intestinal microbiome associated with the degradation of certain polysaccharides, such as chitin (a component of arthropod exoskeletons and fungal cell walls), has been suggested. This is likely due to the reduced consumption of insects in modern diets [16]. Taken together, these findings suggest adaptive changes in the human microbiome over time due to modifications in diet and the environment [17].

Despite the information generated from ancient microbiome studies, it is still necessary to continue collecting data that sheds light on changes in microbial communities and the functional profiles of the ancient human microbiome. In this sense, understanding both specific changes (at the population or regional level) and broader patterns (at the species level) throughout history is crucial in order to understand the relationship between humans and their microbiome, as well as the health status of past human populations [18].

In this context, this study aims to contribute to the expansion of knowledge about the ancient human microbiome through the characterization of bacterial communities present in paleofeces and mummified tissue samples from a pre-Hispanic individual found in El Saucillo, Zimapán, Hidalgo. The samples were provided by La Coordinación Nacional de Conservación del Patrimonio Cultural-INAH [19,20]. The individual was a young adult male, aged between 21 and 35 years at the time of death, discovered in a rock shelter in El Saucillo, within a mortuary bundle lying on a cultural organic layer composed mainly of grass and xerophytic vegetation [21]. The young adult likely belonged to the Otopame culture [22], one of the oldest cultures in Mesoamerica. While previous studies on mummies and human remains have been conducted, the challenge of finding such samples hinders a complete reconstruction of the ancient microbiome and its correlation with the lifestyle of the inhabitants. The first analysis of the ancient microbiome in mummies was carried out on an individual from the ancient Inca culture in Peru [15], followed by the description of the intestinal tissue of a male found in the glaciers of Germany, known as the Tyrolean Iceman dated to the Neolithic era (5350–5100 BP) [11].

For the analysis of the ancient microbiome, DNA extraction and subsequent sequencing of the 16S rRNA gene from the paleofeces and intestinal tissue were performed using the Illumina MiSeq platform. This initial characterization successfully addressed the challenge of identifying some bacterial groups that may have been part of the Zimapán man’s gut microbiome. All these findings contribute to the continuous expansion of knowledge regarding the ancient microbiome across various time frames, encompassing diverse historical, geographical, and environmental contexts.

## Methods

### Paleofeces and mummified tissue samples

**Fig 1:**
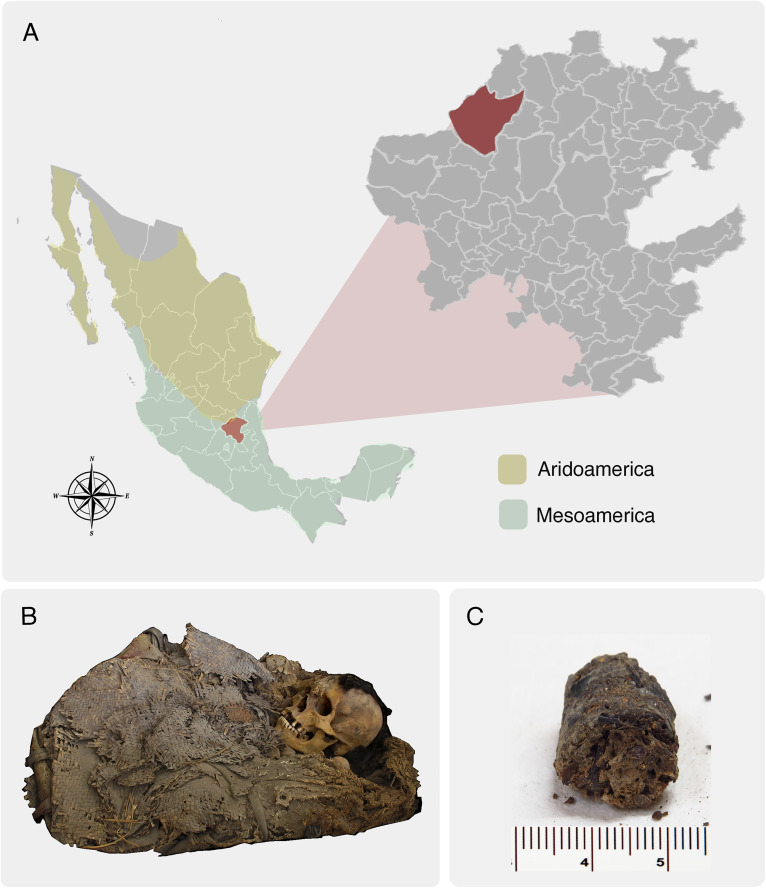
Remains of the Zimapán Man and Location Where He Was Found. Map of Mexico highlighting in red the state of Hidalgo and the locality of Zimapán, where the individual was recovered. Brown and green shading indicate the regions of Aridoamerica (brown) and Mesoamerica (green), B) Mortuary bundle and remains of the Zimapán individual, and C) Samples of paleofeces from the Zimapán individual.

In 2014, residents of El Saucillo, Zimapán, Hidalgo, reported the discovery of a mortuary bundle to the Instituto Nacional de Antropología e Historia (INAH) [19]. The mortuary bundle was located in a rock shelter where both environmental and microenvironmental conditions favored the exceptional preservation of bone tissue (The state of conservation is described in detail in S1 File). Additionally, remnants of soft tissue, including skin, fragments of the intestinal wall, fecal remains, and blood vessels, were also found in the mortuary bundle (Fig. 1) [21]. Sampling of the intestinal wall and fecal remains was conducted in a sterile setting and placed into sterile Whirl Pak® bags. The samples were stored in a biological archive at the Laboratorio de Conservación, Restauración e Investigación de Patrimonio de Origen Orgánico de la Coordinación Nacional de Conservación del Patrimonio Cultural- INAH. The individual was dated using 14C to 936 years BP, placing it in the early Postclassic period of the archaeological chronology of central Mexico (900 AD – 1200 AD).

### Laboratory conditions

The extraction of ancient DNA was carried out at the Laboratorio de Genómica Bacteriana, Facultad de Medicina, UNAM. The protocol followed the recommendations of Knapp et al., 2011 [23], including the establishment of permanent sterile areas. One room was exclusively designated for changing laboratory attire, while an adjacent room was used solely for sample handling and DNA extraction. Both areas were equipped with UV lamps and air-conditioning units fitted with UV light, which were activated 3 hours before and after an extraction event. All materials used were single-use and underwent a decontamination process through autoclaving and subsequent exposure to UV light before DNA extraction. Access to the ancient DNA extraction area required wearing a disposable surgical overall and plastic booties. DNA extraction and amplification were first carried out in our specialized laboratory for handling ancient material. A second replicate was conducted in a separate laboratory located approximately 10 km away. Although this second laboratory was not specifically designed according to the specifications of Knapp et al. (2011) [23], the same handling guidelines were strictly followed.

### Sample preparation

Approximately 600 mg of paleofeces were weighed, and the internal content was extracted to eliminate the external layer and minimize the risk of surface contamination. The internal material was then pulverized using a disposable mortar, and the resulting powder was homogenized. Between 150 and 200 mg of this material were weighed per sample. In total, two samples (S1 and S2) were obtained for DNA extraction.

For the mummified intestinal tissue, a 250 mg sample was used. The tissue was carefully cleaned with a 3% bleach solution, allowed to dry, and then processed for DNA extraction. Two independent DNA extractions were performed from the same sample in two separate laboratories, resulting in extracts I3 and I3.2.

### Ancient DNA extraction

The samples were treated with 300 µL of lysis buffer (containing 400 mM NaCl, 10 mM Tris-HCl pH 7.5, 2 mM Na₂EDTA pH 8.2, and 20% SDS) and incubated at room temperature with agitation (250 rpm) overnight. Subsequently, 4 µL of Proteinase K (20 mg/mL) was added to each tube, followed by incubation at 65 °C for three hours. After incubation, the samples were centrifuged at 4000 rpm for 10 minutes, and the resulting supernatant was transferred to a new tube. Then, 100 µL of 5 M NaCl was added to each tube, and incubation continued for 30 minutes at −70 °C. The samples were centrifuged again at 4000 rpm for 10 minutes, and the supernatant was transferred to a clean tube. Next, 150 µL of isopropanol was added, and the samples were incubated at room temperature for 12 hours. A final centrifugation at 4000 rpm for 10 minutes was performed. The resulting DNA pellet was washed twice with 70% ethanol, air-dried, and rehydrated in 100 µL of ultrapure water. Finally, to remove excess salts that may have co-precipitated with the DNA, the extract was cleaned and concentrated using the DNeasy PowerSoil Kit (Qiagen), following the manufacturer’s instructions. A negative control (C1) was included in the extraction process.

### 16S rRNA sequencing

After confirming the presence of DNA through 2% agarose gel electrophoresis and quantification using NanoDrop spectrophotometry, the 16S rRNA gene was sequenced. Two independent sequencing runs were performed using samples from the same individuals but derived from different DNA extraction events. These runs were carried out to validate the results across two distinct hypervariable regions of the 16S rRNA gene.

In the first run, the V3 hypervariable region (U341F/534R) was amplified and sequenced from two paleofeces samples (S1 and S2), one intestinal tissue sample (I3), and a negative control (C1). Amplification and sequencing were conducted at the Laboratorio de Secuenciación Masiva at IBT, UNAM.

In the second run, DNA was independently extracted from the same intestinal tissue (I3.2), along with a negative control (C2), and the V3–V4 region (341F/805R) was amplified and sequenced at the Integrated Microbiome Resource (IMR) in Canada. Negative controls were included in both sequencing runs to monitor potential contamination.

### 16S rRNA sequence processing

Sequence data processing was conducted using QIIME2 (version 2022.2) [24]. Each sequencing dataset, defined by the utilization of either v3 or v3-v4 primer pairs, was processed independently. Adapter removal was performed using the Cutadapt program [25], and sequence quality was assessed using QIIME2 native plugins (quality-filter q-score) [26]. Sequence merging, chimera removal, OTU generation, and clustering were executed using the VSEARCH pipeline [27]. Clustering was performed against the SILVA 138 database (99% reference), using a 97% similarity threshold. Taxonomic assignment was conducted with a scikit-learn classifier trained on the SILVA 138 database [28,29].

Although USEARCH has been recommended as the optimal tool for analyzing ancient microbiome 16S rRNA data [30], it is no longer supported in QIIME2. Therefore, VSEARCH was selected as a suitable alternative due to its similar clustering strategy [27]. Finally, a phylogenetic tree was constructed using FastTree [31]. All software was run with default parameters.

### Environmental 16S rRNA sequences used for comparison with the zimapán man’s microbial communities

To compare the Zimapán individual’s samples with other microbiome sources, 16S rRNA gene sequences were retrieved from various environments. These included microbiomes from soils in the state of Hidalgo [32], compost [33], the modern human gut microbiome derived from fecal samples representing populations with hunter-gatherer, agricultural, agropastoral, and urban lifestyles [34–36], as well as samples from the contemporary human colon [37]. Additionally, ancient microbiome data from mummified Andean colons and coprolites from the Middle Ages in Namur (Belgium) were included [12,38]. Accession numbers of all datasets are listed in S1 Table.

Processing of these external sequences was conducted separately using QIIME2 (version 2022.2). The resulting feature tables were then merged with the Zimapán dataset into a single file for downstream analysis in R [39].

### Data analysis

To analyze the relative abundance of microbial groups from the Zimapán samples and examine abundance patterns across different taxonomic levels, relative abundance tables (abundance > 0.001) were generated using the phyloseq package in R [40]. Multivariate analyses were conducted to compare microbial composition among Zimapán samples and sequences obtained from various environments, including soil, compost, modern and ancient human intestinal microbiomes. Principal Coordinates Analysis (PCoA) with unweighted UniFrac distance at the genus and family levels were performed using the phyloseq package [40]. All plots were generated with the ggplot2 library in R [41].

Furthermore, the contribution of microbiomes from various modern sources (soil, compost, and modern human microbiome) to the Zimapán samples was estimated using the SourceTracker2 program [42]. For this analysis, we integrated the previously described datasets, excluding ancient samples (Namur Middle Ages coprolites and the mummified Andean colon). In addition, we defined a “non-industrialized” category that included populations with traditional lifestyles (agropastoralists, hunter-gatherers, and rural farming communities). The analysis was performed using OTU tables at the family level, applying the program’s default parameters.

## Results

### DNA extraction and sample processing

Following the previously described DNA extraction protocol, DNA concentrations ranging from 200 to 400 ng/µl were successfully achieved. Notably, sample S1 exhibited the highest concentration, while S2 showed the lowest. Remarkably, the negative control yielded a DNA concentration of 6.2 ng/µl.

Regarding the number of reads obtained in each 16S rRNA gene sequencing event, the first event (primers U341F/534R) resulted in 510,740 reads for S1, 40,210 for S2, and 2,860 reads for I3. Conversely, the second sequencing event (primers 341F-805R) produced a total of 552,371 reads for sample I3.2. After sequence denoising and quality control processing, the retained sequence percentages were 99%, 99%, 99%, and 89% for samples S1, S2, I3, and I3.2, respectively.

### Relative abundances of bacterial groups

**Fig 2:**
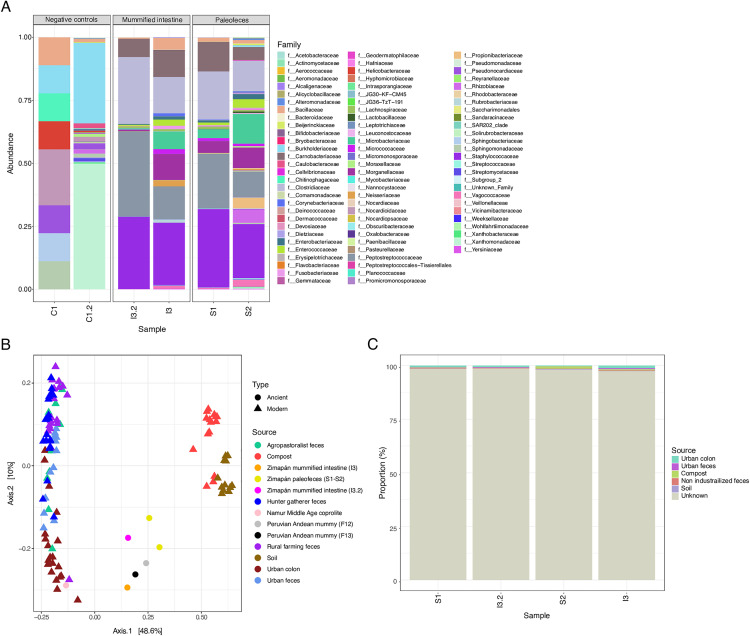
Bacterial composition of the Zimapán samples at the family level and comparison with different environmental sources using multivariate analysis and SourceTracker. A) Stacked bar plot showing the abundance of bacterial families identified in the Zimapán samples (relative abundance > 0.001). The plot includes negative controls (C1 and C2), mummified intestinal tissue of the Zimapán individual (I3 and I3.2), and paleofeces samples from the same individual (S1 and S2), B) Multivariate analysis integrating Zimapán samples with various environmental datasets. Principal Coordinate Analysis (PCoA) at the family level was performed using unweighted UniFrac distances, including samples from soil, compost, ancient gut microbiomes (Namur coprolite, Andean mummy tissue, and Zimapán samples), and modern gut microbiomes from populations with different lifestyles (hunter-gatherers, agropastoralists, and urban populations). Circles indicate ancient samples, while triangles indicate modern samples, and C) SourceTracker analysis of the Zimapán samples. The analysis included samples from soils, compost, gut microbiomes of urban populations (colon and feces), and gut microbiomes from non-industrialized populations (agropastoralists, hunter-gatherers, and rural farming populations).

To analyze the bacterial community composition within the paleofeces and mummified intestinal tissue samples from the Zimapán individual, relative abundance tables at the family and genus levels were generated (Fig 2A). Across all Zimapán samples (S1, S2, I3, and I3.2), consistent patterns were observed, with prevalent bacterial families including *Peptostreptococcaceae*, *Clostridiaceae*, *Staphylococcaceae*, *Morganellaceae*, *Enterobacteriaceae*, *Enterococcaceae*, *Bacillaceae*, *Carnobacteriaceae*, and *Lachnospiraceae*. In contrast, the negative control samples (C1 and C2) exhibited distinct compositional profiles compared to the ancient samples. Notably, *Nocardioidaceae* dominated in C1, while *Xanthomonadaceae* was the most abundant group in C2 (Fig 2A)

Moreover, stacked bar plots at the genus level showed patterns similar to those observed at the family level (Supp. Fig 1A). The most abundant genera included *Jeotgalicoccus*, *Clostridium sensu stricto*, *Atopostipes*, *Enterococcus*, *Escherichia−Shigella*, and *Providencia*. Interestingly, sample I3.2 (mummified intestine) exhibited higher abundances of additional genera such as *Romboutsia* and *Terrisporobacter*. In contrast, the negative controls showed bacterial compositions distinct from those of the Zimapán samples (Supp. Fig 1A).

### Multivariate analysis integrating microbiomes from different environments

In the multivariate analysis, we integrated 16S rRNA gene sequences from soil, compost, the human gut microbiome (feces and colon), and ancient microbiomes (mummified colon and coprolites), along with the samples from the Zimapán individual (Fig 2B). The objective was to visualize differences and similarities between the microbial communities identified in this study and those from other environments.

Results from the PCoA using unweighted UniFrac distance at the family level revealed a strong similarity between the Zimapán samples (S1, S2, I3, and I3.2) and colon samples from the Pre-Columbian Andean mummies (Fig 2B). Notably, the Zimapán samples clustered between soil and human intestinal microbiome samples. In particular, the intestinal tissue sample I3.2 was the closest to those from the modern human colon microbiome. In contrast, the Namur Middle Ages coprolite clustered more closely with samples from the modern human gut microbiome. Importantly, the PCoA at the genus level showed similar patterns (Supp. Fig 1B).

### Sourcetracker analysis

The SourceTracker analysis was performed using 16S rRNA gene sequences from soils, compost, and the contemporary human gut microbiome (colon and feces) from populations with different lifestyles, along with the Zimapán individual’s samples (Fig 2C; S1 Table). The objective was to estimate the proportion of microbial contributions from these different environments to the microbiome of the Zimapán samples. In this analysis, more than 90% of the microbial composition in the Zimapán samples could not be assigned to any of the included source environments. Among the identifiable sources, the highest contribution corresponded to the human gut microbiome from colon and fecal samples, followed by the compost microbiome (Fig 2C).

## Discussion

In this study, we analyzed the microbial composition of paleofeces and intestinal tissue from an individual dating back to the Postclassic period, situated in Zimapán, Hidalgo. This region lies on the border between Mesoamerica and Aridoamerica. Mesoamerica played a crucial role in domestication and the rise of major civilizations and cities. In contrast, the Aridoamerica region is known for its sparse human settlements, predominantly inhabited by seasonal seminomadic people who practiced hunting and gathering [43,44].

Through DNA extraction and subsequent 16S rRNA gene sequencing of paleofeces and intestinal tissue from the Zimapán man, various bacterial groups were identified. Although the most abundant group in the samples was a halotolerant microbe (*Jeotgalicoccus*), typically associated with marine soils and sediments [45], other identified groups are potentially related to the human gut microbiome. Among the most abundant taxa, we identified bacteria from the families *Peptostreptococcaceae*, *Clostridiaceae*, *Enterobacteriaceae*, *Enterococcaceae*, *Morganellaceae*, and *Lachnospiraceae* (Fig 2A).

The *Peptostreptococcaceae* family includes fermentative bacteria considered commensals of the human gut, contributing to intestinal homeostasis [46]. Notably, *Romboutsia hominis* (*Peptostreptococcaceae* family) was detected in one of the analyzed samples (I3.2) (Supp. Fig 1A). This species is the only known member of the *Romboutsia* genus that have been recently isolated from the human gut [47]. Although little is known about its functional roles, *R. hominis* has been associated with carbohydrate utilization and the biosynthesis of vitamins and other cofactors [47].

A high abundance of bacteria from the *Clostridium* genus was found in the Zimapán samples, consistent with previous reports from colon samples of pre-Columbian Andean mummies [48]. *Clostridium* is known to colonize the human intestine, performing a wide range of metabolic functions. Various *Clostridium* species are capable of utilizing nutrients (such as complex polysaccharides) that the human host cannot digest, producing short-chain fatty acids (SCFAs) [49]. SCFAs play a fundamental role in intestinal homeostasis [49].

A study conducted in 2021 by Wibowo et al [16] on paleofeces and coprolites revealed a broad range of metabolic functions in the *Clostridiaceae* family, including the degradation of carbohydrate-active enzymes (CAZymes). Particularly, chitin CAZymes were identified, possibly associated with insect consumption in ancient populations. In the Zimapán samples, a high abundance of the *Clostridium* genus was identified, suggesting a potential role in insect degradation. This is particularly relevant given that the Zimapán man was recovered in the state of Hidalgo, Mexico, a region with a long-standing cultural tradition of entomophagy (insect consumption) [50].

*Lachnospiraceae*, alongside *Clostridiaceae*, exhibited high abundance in the Zimapán samples. *Lachnospiraceae* is recognized as a core member of the human gut microbiome and is associated with the production of short-chain fatty acids (SCFAs) [51]. Within this family, certain species contribute to the breakdown of cellulose and hemicellulose, polysaccharides found in plant cell walls that humans are unable to digest. The activity of these polysaccharide-degrading bacteria enhances the bioavailability of otherwise inaccessible dietary components for the host [51]. The prevalence of such taxa suggests that the Zimapán individual may have sustained a diverse, plant-based diet, likely including agaves, yuccas, mesquites, and prickly pears, among other species characteristic of xerophilic environments [52].

Other groups from the *Enterobacteriaceae* and *Enterococcaceae* families were also identified. These groups are common members of the gastrointestinal microbiota in humans and other vertebrates. However, under conditions of intestinal dysbiosis, these groups can transition into pathobionts [53,54].

Beyond that, the multivariate analysis revealed a close similarity between the Zimapán samples and the pre-Columbian Andean mummies. Although the Zimapán samples clustered near the modern human gut microbiome samples, they didn’t share the same space in the PCoA. That could suggest a slight deviation from the human gut microbiota composition. This arrangement in the multivariate analysis is likely attributed to the presence of non-intestinal bacterial taxa in the samples from the Zimapán individual, potentially introduced through the gradual accumulation of environmental bacteria over time. Despite meticulous efforts to remove the outer layer of the paleofeces and clean the surface of the mummified intestinal tissue, soil and sediment associated groups such as *Jeotgalicoccus*, *Sphingomonadaceae*, *Vagococcaceae* were detected. All these factors could have influenced the microbial composition, subsequently impacting the distribution of the Zimapán samples in the multivariate analysis, resulting in a slight separation of the samples representing the human gut microbiota. These findings align with those observed in the SourceTracker2 analysis. Among all the environments included in the analysis, the human gut microbiome accounted for the largest proportion of the microbial community in the Zimapán samples. However, it is noteworthy that a substantial fraction of the microbial composition could not be assigned to any known source.

Finally, it is important to highlight that, despite detecting representative groups of the human gut microbiome such as *Bacteroidaceae* and *Bifidobacteriaceae*, were found in very low abundance, while others, including *Prevotellaceae* and *Ruminococcaceae*, were not identified in the Zimapán samples. These groups are typically abundant in the modern human microbiome and have also been reported in previous ancient gut microbiome studies [14,16]. These results suggest that alternative approaches, such as metagenomics, may be necessary to confirm the presence of these bacteria and verify their persistence in ancient samples. It has been proposed that metagenomic approaches are more appropriate when working with highly fragmented DNA, as is characteristic of ancient samples [55]. Indeed, several studies have shown that ancient DNA often consists of fragments approximately 40 base pairs in length [56], which can limit the effectiveness of 16S rRNA gene-based methods. Moreover, metagenomics could provide a more accurate view of ancient DNA damage patterns in the bacterial taxa detected in the Zimapán samples [56]. Nevertheless, conducting an initial characterization using 16S rRNA sequencing was a necessary first step to identify potential members of the ancient gut microbiome. This approach sets the foundation for deeper investigations, including taxonomic and functional characterization and the reconstruction of ancient bacterial genomes, as has been successfully achieved in other ancient microbiome studies [13,16,17].

## Conclusions

The integration of anthropological, archaeological, and microbiome data serves as a powerful tool for unraveling human evolution and its intricate relationship with both biological and social environments. In this study, we present the first characterization of the gut microbiome of an individual from Zimapán, Hidalgo, using high-throughput sequencing of the 16S rRNA gene from paleofeces and mummified tissue. This approach enabled the identification of diverse bacterial groups, including taxa recognized as potential members of the human intestinal microbiome, such as *Romboutsia hominis*, a species exclusively isolated from the human gut and, until now, unreported in the ancient human gut microbiome. This finding raises the possibility that the Zimapán paleofeces could preserve authentic ancient gut bacteria. Conversely, the absence of other representative and typically abundant human-associated taxa underscores the need for complementary strategies to further explore the Zimapán samples. These efforts could also include functional analyses of the gut microbiome of this individual. Despite these limitations, the primary objective of identifying gut microbiome components in a seasonal seminomadic hunter-gatherer individual was achieved, thereby contributing to the ongoing expansion of knowledge about the ancient human microbiome across diverse temporal and cultural contexts.

## Supporting information

S1 Fig Bacterial composition of the Zimapán samples at the genus level. A) Stacked bar plot showing the abundance of bacterial genera identified in the Zimapán samples (relative abundance > 0.001). The plot includes negative controls (C1 and C2), mummified intestinal tissue of the Zimapán individual (I3 and I3.2), and paleofeces samples from the same individual (S1 and S2) and, B) Multivariate analysis integrating Zimapán samples with different environmental datasets. Principal Coordinate Analysis (PCoA) at the genus level was performed using unweighted UniFrac distances, including samples from soil, compost, ancient gut microbiomes (Namur coprolite, Andean mummy tissue, and Zimapán samples), and modern gut microbiomes from populations with different lifestyles (hunter-gatherers, agropastoralists, and urban populations). Circles indicate ancient samples, while triangles indicate modern samples.
(TIF)

S1 Table List of accession numbers for all sequences included in the analyses. The table includes sequences downloaded from public databases for comparison with Zimapán samples.
(CSV)

S1 File Detailed description of the preservation state of the mortuary bundle from El Saucillo, Zimapán. (DOCX)

## References

[1] Lan T, Lindqvist C. Technical advances and challenges in genome-scale analysis of ancient DNA. In: Lindqvist C, Rajora OP, editors. Paleogenomics: genome-scale analysis of ancient DNA. Berlin: Springer. 2019. p. 3–29.

[2] Brownstein KJ, Tushingham S, Damitio WJ, Nguyen T, Gang DR. An ancient residue metabolomics-based method to distinguish use of closely related plant species in ancient pipes. Front Mol Biosci. 2020;7:133. doi: 10.3389/fmolb.2020.00133 32671097 PMC7332879

[3] Nodari R, Drancourt M, Barbieri R. Paleomicrobiology of the human digestive tract: A review. Microb Pathog. 2021;157:104972. doi: 10.1016/j.micpath.2021.104972 34029658

[4] Hodgson JA, Disotell TR. Anthropological genetics: inferring the history of our species through the analysis of DNA. Evo Edu Outreach. 2010;3(3):387–98. doi: 10.1007/s12052-010-0262-9

[5] Stone AC, Ozga AT. Ancient DNA in the study of ancient disease.In: Buikstra JE, editor. Ortner’s Identification of Pathological Conditions in Human Skeletal Remains. Elsevier. 2019. p. 183–210. doi: 10.1016/b978-0-12-809738-0.00008-9

[6] Reinhard KJ, Vaughn B. Coprolite analysis: a biological perspective on archaeology. In: Schiffer MB, editor. Archaeological method and theory. New York: University of Arizona Press. 1992. p. 245–88.

[7] Warinner C, Speller C, Collins MJ, Lewis CM Jr. Ancient human microbiomes. J Hum Evol. 2015;79:125–36. doi: 10.1016/j.jhevol.2014.10.016 25559298 PMC4312737

[8] Lloyd-Price J, Abu-Ali G, Huttenhower C. The healthy human microbiome. Genome Med. 2016;8(1):51. doi: 10.1186/s13073-016-0307-y 27122046 PMC4848870

[9] Jandhyala SM, Talukdar R, Subramanyam C, Vuyyuru H, Sasikala M, Nageshwar Reddy D. Role of the normal gut microbiota. World J Gastroenterol. 2015;21(29):8787–803. doi: 10.3748/wjg.v21.i29.8787 26269668 PMC4528021

[10] Rosas-Plaza S, Hernández-Terán A, Navarro-Díaz M, Escalante AE, Morales-Espinosa R, Cerritos R. Human gut microbiome across different lifestyles: from hunter-gatherers to urban populations. Front Microbiol. 2022;13:843170. doi: 10.3389/fmicb.2022.843170 35558108 PMC9087276

[11] Cano RJ, Tiefenbrunner F, Ubaldi M, Del Cueto C, Luciani S, Cox T, et al. Sequence analysis of bacterial DNA in the colon and stomach of the Tyrolean Iceman. Am J Phys Anthropol. 2000;112(3):297–309. doi: 10.1002/1096-8644(200007)112:3<297::AID-AJPA2>3.0.CO;2-0 10861348

[12] Santiago-Rodriguez TM, Fornaciari G, Luciani S, Dowd SE, Toranzos GA, Marota I, et al. Taxonomic and predicted metabolic profiles of the human gut microbiome in pre-Columbian mummies. FEMS Microbiol Ecol. 2016;92(11):fiw182. doi: 10.1093/femsec/fiw182 27559027

[13] Santiago-Rodriguez TM, Narganes-Storde YM, Chanlatte L, Crespo-Torres E, Toranzos GA, Jimenez-Flores R, et al. Microbial communities in pre-columbian coprolites. PLoS One. 2013;8(6):e65191. doi: 10.1371/journal.pone.0065191 23755194 PMC3673975

[14] Tito RY, Knights D, Metcalf J, Obregon-Tito AJ, Cleeland L, Najar F, et al. Insights from characterizing extinct human gut microbiomes. PLoS One. 2012;7(12):e51146. doi: 10.1371/journal.pone.0051146 23251439 PMC3521025

[15] Ubaldi M, Luciani S, Marota I, Fornaciari G, Cano RJ, Rollo F. Sequence analysis of bacterial DNA in the colon of an Andean mummy. Am J Phys Anthropol. 1998;107(3):285–95. doi: 10.1002/(sici)1096-8644(199811)107:3<285::aid-ajpa5>3.0.co;2-u 9821493

[16] Wibowo MC, Yang Z, Borry M, Hübner A, Huang KD, Tierney BT, et al. Reconstruction of ancient microbial genomes from the human gut. Nature. 2021;594(7862):234–9. doi: 10.1038/s41586-021-03532-0 33981035 PMC8189908

[17] Rifkin RF, Vikram S, Ramond J-B, Rey-Iglesia A, Brand TB, Porraz G, et al. Multi-proxy analyses of a mid-15th century Middle Iron Age Bantu-speaker palaeo-faecal specimen elucidates the configuration of the “ancestral” sub-Saharan African intestinal microbiome. Microbiome. 2020;8(1):62. doi: 10.1186/s40168-020-00832-x 32375874 PMC7204047

[18] Sonnenburg ED, Sonnenburg JL. The ancestral and industrialized gut microbiota and implications for human health. Nat Rev Microbiol. 2019;17(6):383–90. doi: 10.1038/s41579-019-0191-8 31089293

[19] Galán Tamés M, Mainou L, Gómez González J, Aguilar Romero A. The mathematical pattern of the shroud in the mortuary bundle of Zimapán, Hidalgo. RevLatEtnomat. 2021;14(2):32–53. doi: 10.22267/relatem.21142.84

[20] Gómez González J, Mainou L, Aguilar Romero A, González Hernández G, Beramendi Orosco LE, Straulino Mainou L, et al. Arqueometría aplicada a la conservación de textiles arqueológicos de fibras celulósicas. Petate y mortaja de un fardo mortuorio de Zimapán, Hidalgo. BSGM. 2019;71(2):429–44. doi: 10.18268/bsgm2019v71n2a12

[21] Mainou L, Aguilar-Romero A, Gómez-González J, Villa-Sánchez G, Gómez-Valdés JA. Restos de sangre que evidencian la muerte de un individuo por hemorragia. Fardo mortuorio de El Saucillo, Zimapán, Hidalgo. Estudios sobre conservación restauración y museología. Ciudad de México: ENCRyM. 2017.

[22] Acuña R. Relación de las Minas de Zimapán. Relaciones geográficas del siglo XVI: México. Ciudad de México: Universidad Nacional Autónoma de México. 1985.

[23] Knapp M, Clarke AC, Horsburgh KA, Matisoo-Smith EA. Setting the stage - building and working in an ancient DNA laboratory. Ann Anat. 2012;194(1):3–6. doi: 10.1016/j.aanat.2011.03.008 21514120

[24] Caporaso JG, Kuczynski J, Stombaugh J, Bittinger K, Bushman FD, Costello EK, et al. QIIME allows analysis of high-throughput community sequencing data. Nat Methods. 2010;7(5):335–6. doi: 10.1038/nmeth.f.303 20383131 PMC3156573

[25] Martin M. Cutadapt removes adapter sequences from high-throughput sequencing reads. EMBnet j. 2011;17(1):10. doi: 10.14806/ej.17.1.200

[26] Bokulich NA, Subramanian S, Faith JJ, Gevers D, Gordon JI, Knight R, et al. Quality-filtering vastly improves diversity estimates from Illumina amplicon sequencing. Nat Methods. 2013;10(1):57–9. doi: 10.1038/nmeth.2276 23202435 PMC3531572

[27] Rognes T, Flouri T, Nichols B, Quince C, Mahé F. VSEARCH: a versatile open source tool for metagenomics. PeerJ. 2016;4:e2584. doi: 10.7717/peerj.2584 27781170 PMC5075697

[28] Yilmaz P, Parfrey LW, Yarza P, Gerken J, Pruesse E, Quast C, et al. The SILVA and “All-species Living Tree Project (LTP)” taxonomic frameworks. Nucleic Acids Res. 2014;42(Database issue):D643-8. doi: 10.1093/nar/gkt1209 24293649 PMC3965112

[29] Pedregosa F, Varoquaux G, Gramfort A, Michel V, Thirion B, Grisel O, et al. Scikit-learn: machine learning in python. J Mach Learn Res. 2011;12:2825–30. doi: 10.5555/1953048.2078195

[30] Velsko IM, Frantz LAF, Herbig A, Larson G, Warinner C. Selection of Appropriate Metagenome Taxonomic Classifiers for Ancient Microbiome Research. mSystems. 2018;3(4):e00080-18. doi: 10.1128/mSystems.00080-18 30035235 PMC6050634

[31] Price MN, Dehal PS, Arkin AP. FastTree 2--approximately maximum-likelihood trees for large alignments. PLoS One. 2010;5(3):e9490. doi: 10.1371/journal.pone.0009490 20224823 PMC2835736

[32] Lüneberg K, Schneider D, Siebe C, Daniel R. Drylands soil bacterial community is affected by land use change and different irrigation practices in the Mezquital Valley, Mexico. Sci Rep. 2018;8(1):1413. doi: 10.1038/s41598-018-19743-x 29362388 PMC5780513

[33] Pot S, De Tender C, Ommeslag S, Delcour I, Ceusters J, Gorrens E, et al. Understanding the shift in the microbiome of composts that are optimized for a better fit-for-purpose in growing media. Front Microbiol. 2021;12:643679. doi: 10.3389/fmicb.2021.643679 33897654 PMC8059793

[34] Quagliariello A, Di Paola M, De Fanti S, Gnecchi-Ruscone GA, Martinez-Priego L, Pérez-Villaroya D, et al. Gut microbiota composition in Himalayan and Andean populations and its relationship with diet, lifestyle and adaptation to the high-altitude environment. J Anthropol Sci. 2019;96:189–208. doi: 10.4436/JASS.97007 31782749

[35] Ruggles KV, Wang J, Volkova A, Contreras M, Noya-Alarcon O, Lander O, et al. Changes in the Gut Microbiota of Urban Subjects during an Immersion in the Traditional Diet and Lifestyle of a Rainforest Village. mSphere. 2018;3(4):e00193-18. doi: 10.1128/mSphere.00193-18 30158281 PMC6115531

[36] Smits SA, Leach J, Sonnenburg ED, Gonzalez CG, Lichtman JS, Reid G, et al. Seasonal cycling in the gut microbiome of the Hadza hunter-gatherers of Tanzania. Science. 2017;357(6353):802–6. doi: 10.1126/science.aan4834 28839072 PMC5891123

[37] Ryan FJ, Ahern AM, Fitzgerald RS, Laserna-Mendieta EJ, Power EM, Clooney AG, et al. Colonic microbiota is associated with inflammation and host epigenomic alterations in inflammatory bowel disease. Nat Commun. 2020;11(1):1512. doi: 10.1038/s41467-020-15342-5 32251296 PMC7089947

[38] Appelt S, Armougom F, Le Bailly M, Robert C, Drancourt M. Polyphasic analysis of a middle ages coprolite microbiota, Belgium. PLoS One. 2014;9(2):e88376. doi: 10.1371/journal.pone.0088376 24586319 PMC3938422

[39] Oksanen J, Kindt R, Legendre P, O’Hara B, Simpson GL, Solymos PM, et al. The vegan package. Community Ecology Package. 2008. p. 1–182.

[40] McMurdie PJ, Holmes S. phyloseq: an R package for reproducible interactive analysis and graphics of microbiome census data. PLoS One. 2013;8(4):e61217. doi: 10.1371/journal.pone.0061217 23630581 PMC3632530

[41] Villanueva RAM, Chen ZJ. ggplot2: Elegant Graphics for Data Analysis (2nd ed.). Measurement: Interdisciplinary Research and Perspectives. 2019;17(3):160–7. doi: 10.1080/15366367.2019.1565254

[42] Knights D, Kuczynski J, Charlson ES, Zaneveld J, Mozer MC, Collman RG, et al. Bayesian community-wide culture-independent microbial source tracking. Nat Methods. 2011;8(9):761–3. doi: 10.1038/nmeth.1650 21765408 PMC3791591

[43] Mellink E, Riojas-López ME, Rivera-Villanueva JA. Reconsideration of the nomadic condition of the southernmost Guachichiles based on the relationship with their environment. J Ethnobiol Ethnomed. 2018;14(1):24. doi: 10.1186/s13002-018-0223-x 29609628 PMC5880072

[44] Taylor WW. The hunter-gatherer nomads of Northern Mexico: a comparison of the archival and archaeological records. World Archaeol. 1972;4(2):167–78.

[45] Liu Z-X, Chen J, Tang S-K, Zhang Y-Q, He J-W, Chen Q-H, et al. Jeotgalicoccus nanhaiensis sp. nov., isolated from intertidal sediment, and emended description of the genus Jeotgalicoccus. Int J Syst Evol Microbiol. 2011;61(Pt 9):2029–34. doi: 10.1099/ijs.0.022871-0 20851914

[46] DuPont HL, Suescun J, Jiang Z-D, Brown EL, Essigmann HT, Alexander AS, et al. Fecal microbiota transplantation in Parkinson’s disease-A randomized repeat-dose, placebo-controlled clinical pilot study. Front Neurol. 2023;14:1104759. doi: 10.3389/fneur.2023.1104759 36937520 PMC10019775

[47] Gerritsen J, Hornung B, Ritari J, Paulin L, Rijkers GT, Schaap PJ, et al. A comparative and functional genomics analysis of the genusRomboutsiaprovides insight into adaptation to an intestinal lifestyle. Cold Spring Harbor Laboratory. 2019. doi: 10.1101/845511

[48] Santiago-Rodriguez TM, Fornaciari G, Luciani S, Dowd SE, Toranzos GA, Marota I, et al. Gut Microbiome of an 11th Century A.D. Pre-Columbian Andean Mummy. PLoS One. 2015;10(9):e0138135. doi: 10.1371/journal.pone.0138135 26422376 PMC4589460

[49] Guo P, Zhang K, Ma X, He P. Clostridium species as probiotics: potentials and challenges. J Anim Sci Biotechnol. 2020;11:24. doi: 10.1186/s40104-019-0402-1 32099648 PMC7031906

[50] Ramos-Elorduy J, Pino Moreno JM, Morales de León J. Análisis químico proximal, vitaminas y nutrimentos inorgánicos de insectos consumidos en el Estado de Hidalgo, México. Folia Entomol Mex. 2002;41(1):15–29.

[51] Vacca M, Celano G, Calabrese FM, Portincasa P, Gobbetti M, De Angelis M. The Controversial Role of Human Gut Lachnospiraceae. Microorganisms. 2020;8(4):573. doi: 10.3390/microorganisms8040573 32326636 PMC7232163

[52] Granados-Sánchez D, López-Ríos GF, Hernández-Hernández J. Agricultura nhanñhu-otomí del Valle del Mezquital, Hidalgo. Terra Latinoam. 2004;22(1):117–26.

[53] Krishnamurthy HK, Pereira M, Bosco J, George J, Jayaraman V, Krishna K, et al. Gut commensals and their metabolites in health and disease. Front Microbiol. 2023;14:1244293. doi: 10.3389/fmicb.2023.1244293 38029089 PMC10666787

[54] Repoila F, Le Bohec F, Guérin C, Lacoux C, Tiwari S, Jaiswal AK, et al. Adaptation of the gut pathobiont Enterococcus faecalis to deoxycholate and taurocholate bile acids. Sci Rep. 2022;12(1):8485. doi: 10.1038/s41598-022-12552-3 35590028 PMC9120511

[55] Ziesemer KA, Mann AE, Sankaranarayanan K, Schroeder H, Ozga AT, Brandt BW, et al. Intrinsic challenges in ancient microbiome reconstruction using 16S rRNA gene amplification. Sci Rep. 2015;5:16498. doi: 10.1038/srep16498 26563586 PMC4643231

[56] Dabney J, Meyer M, Pääbo S. Ancient DNA damage. Cold Spring Harb Perspect Biol. 2013;5(7):a012567. doi: 10.1101/cshperspect.a012567 23729639 PMC3685887

