# Supplementary figures and images for: Microbiome characterization of a pre-Hispanic man from Zimapán, Mexico: Insights into ancient gut microbial communities
Source: PLoS One. 2025 Oct 8;20(10):e0331137. doi: 10.1371/journal.pone.0331137 (PMC12507283; doi:10.1371/journal.pone.0331137)

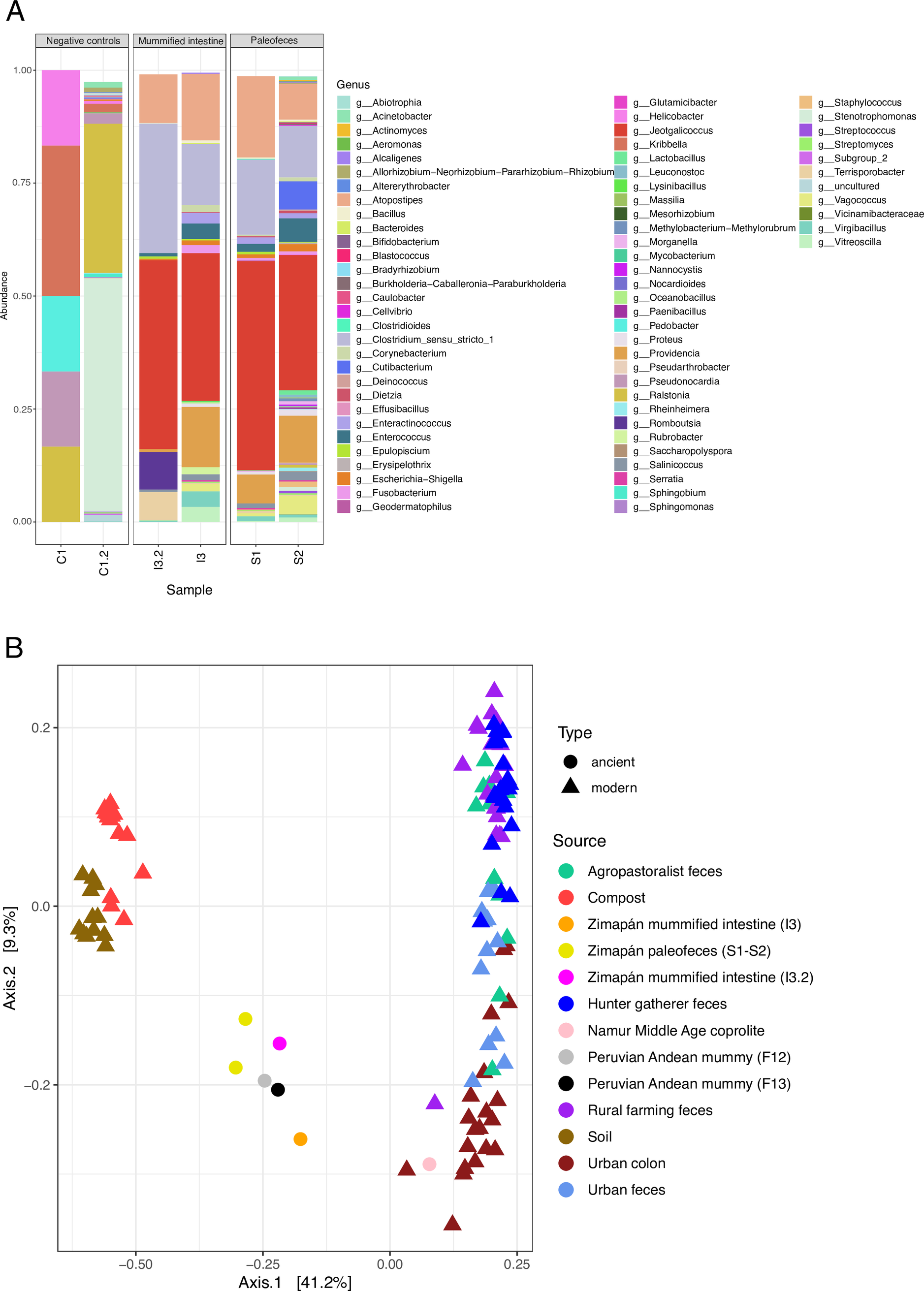

Supplement: S1 Fig — A) Stacked bar plot showing the abundance of bacterial genera identified in the Zimapán samples (relative abundance > 0.001). The plot includes negative controls (C1 and C2), mummified intestinal tissue of the Zimapán individual (I3 and I3.2), and paleofeces samples from the same individual (S1 and S2) and, B) Multivariate analysis integrating Zimapán samples with different environmental datasets. Principal Coordinate Analysis (PCoA) at the genus level was performed using unweighted UniFrac distances, including samples from soil, compost, ancient gut microbiomes (Namur coprolite, Andean mummy tissue, and Zimapán samples), and modern gut microbiomes from populations with different lifestyles (hunter-gatherers, agropastoralists, and urban populations). Circles indicate ancient samples, while triangles indicate modern samples. (TIF) [file pone.0331137.s001.tif]
