# Supplementary material for: Microbiome characterization of a pre-Hispanic man from Zimapán, Mexico: Insights into ancient gut microbial communities
Source: PLoS One. 2025 Oct 8;20(10):e0331137. doi: 10.1371/journal.pone.0331137 (PMC12507283; doi:10.1371/journal.pone.0331137)
Supplement: S1 File — (DOCX) [file pone.0331137.s003.docx]

**Supplementary Information 1**

The individual was wrapped in a mortuary bundle in a hyperflexed left lateral decubitus position. This position was maintained by using two cords: one securing the upper extremities between the thighs and another tying the neck and back of the knees. A 10 cm wide by 240 cm long band made of coyuchi cotton was also used to tie the legs to the pelvic girdle. The body was wrapped in a 244 cm long by 160 cm wide textile, also made of coyuchi cotton. This textile was first tied with two bands, also made of coyuchi cotton, and finally covered with a 156 cm long by 122 cm wide mat. The mat was tied with ropes made of agave fiber. This mortuary bundle was found in a rock shelter located at the top of a massive rock face more than 300 meters high. The shelter is approximately 6 meters deep, 9.4 meters wide, and has an average height of 4.4 meters. It is accessed via a steep path along the top of the mountain range (Fig 1).


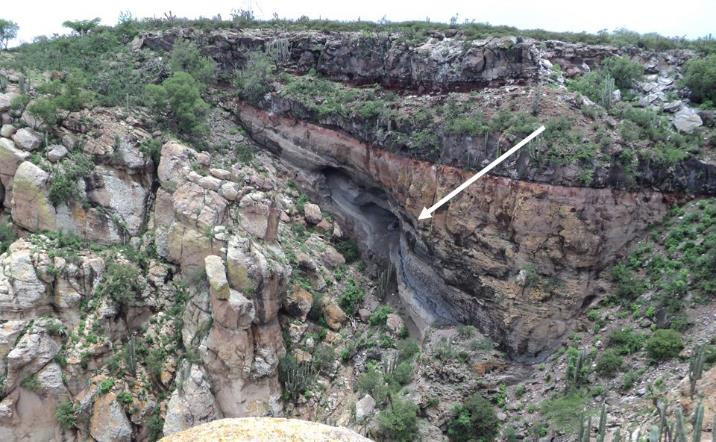


**Figure 1. Location of the rock shelter on a rock face along the north bank of the Zimapán Dam, in the area where the Tula River once flowed.**

Once the Zimapán, Hidalgo bundle was found at the National Coordination for the Conservation of Cultural Heritage (INAH), it was opened and micro-excavated. Tyvek coveralls, nitrile gloves, a face mask, and protective glasses were used for this purpose. First, the top layer of the mat was removed, exposing the shroud and the bands that held it in place. The bands were then untied and the top layer of the textile unwrapped, revealing the individual's skeleton, which was lifted up in anatomical order, starting with the head. Once all the bones were out of the textile, micro-excavation of the sediment began, which consisted of sand, gravel, skin fragments, ligament and tendon fragments, blood vessels, and the coprolite that concerns us today (Figure 2).


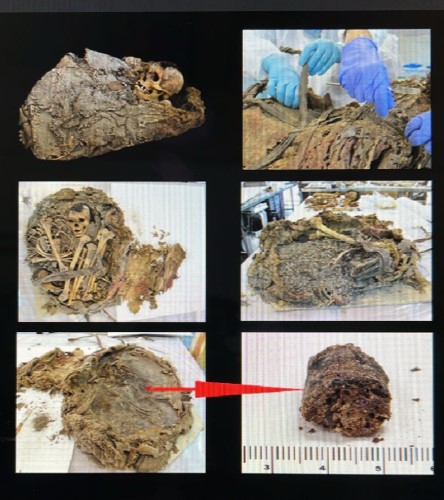


**Figure 2. process of obtaining human intestinal remains including a paleofeces.**
